# Role of histidine decarboxylase gene in the pathogenesis of Tourette syndrome

**DOI:** 10.1002/brb3.2511

**Published:** 2022-02-03

**Authors:** Lulu Xu, Cheng Zhang, Meixiang Zhong, Fengyuan Che, Chengcheng Guan, Xueping Zheng, Shiguo Liu

**Affiliations:** ^1^ Department of Geriatric Medicine The Affiliated Hospital of Qingdao University Qingdao Shandong China; ^2^ Department of Neurology The Eleventh Clinical Medical College of Qingdao University, Linyi People's Hospital Linyi Shandong China; ^3^ Department of Medical Cenetics The Affiliated Hospital of Qingdao University Qingdao Shandong China

**Keywords:** histidine decarboxylase, mouse model, Tourette syndrome, W317X mutation

## Abstract

Tourette syndrome (TS) is caused by complex genetic and environmental factors and is characterized by tics. Histidine decarboxylase (*HDC*) mutation is a rare genetic cause with high penetrance in patients with TS. *HDC*‐knockout (KO) mice have similar behavioral and neurochemical abnormalities as patients with TS. Therefore, *HDC*‐KO mice are considered a valuable TS pathophysiological model as it reveals the underlying pathological mechanisms that cannot be obtained from patients with TS, thus advancing the development of treatment strategies for TS and other tic disorders. This review summarizes some of the recent research hotspots and progress in *HDC*‐KO mice, aiming to deepen our understanding of brain mechanisms relevant to TS. Furthermore, we encapsulate the possible brain nerve cell changes in *HDC*‐KO mice and their potential roles in TS to provide multiple directions for the future research on tics.

## Graphical abstract

Tourette syndrome (TS) is caused by complex genetic and environmental factors and is characterized by tics. Histidine decarboxylase (HDC) mutation is a rare genetic cause with high penetrance in patients with TS. HDC‐knockout (KO) mice have similar behavioral and neurochemical abnormalities as patients with TS. Therefore, HDC‐KO mice are considered a valuable TS pathophysiological model as it reveals the underlying pathological mechanisms that cannot be obtained from patients with TS, thus advancing the development of treatment strategies for TS and other tic disorders. This review summarizes some of the recent research hotspots and progress in HDC‐KO mice, aiming to deepen our understanding of brain mechanisms relevant to TS. Furthermore, we also encapsulate the possible brain nerve cell changes in HDC‐KO mice and their potential roles in TS, to provide multiple directions for the future research on tics.

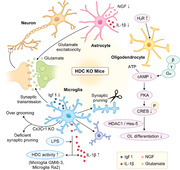

## 1 INTRODUCTION

Tourette syndrome (TS) is a childhood‐onset disorder defined by abnormal developments in the cortico‐subcortical and intracortical neural networks that control motor output and sensory input (Martino et al., 2015). It is associated with congenital dysplasia of the nervous system and is characterized by motor and vocal tics, as well as sensory and cognitive symptoms (Robertson et al., 2009). Tics are sudden, transient, repetitive, and semiautonomous movements or behaviors that help relieve local discomfort or tension. The motor pattern of tics may be divided into simple tics that involve individual muscles or small group of muscles and complex tics that are similar to purposeful voluntary movements associated with more muscles acting in coordination to achieve complex movements (Mink, 2001). Many individuals with tics present associated sensory symptoms, including the constant onset of tic premonitory impulses and a temporary relief thereafter. Tics can be exacerbated by increased stress or fatigue. TS is often associated with high levels of emotional stimulation and stress‐related neurotransmitters or hormones (Leckman, 2002).

TS has a prevalence of 0.3−1%, with a male‐to‐female ratio ranging from 3:1 to 4:1, and the majority of cases start between the ages of 4 and 13 (Baldan et al., 2014; Leckman, 2002). The disease peaks at adolescence, with many patients showing a reduction in tic severity by the age of 18. However, the most severe cases present extreme forms, including shouting obscenities, hitting, or biting, and occur in adulthood. TS has high comorbidity rates with other neurodevelopmental disorders of childhood, such as obsessive‐compulsive disorder (OCD) and attention deficit hyperactivity disorder (ADHD) (Leckman, 2002). Copy number variation (CNV) analysis supports the genetic commonality between TS and autistic spectrum disorders (ASD) (Fernandez et al., 2012). Furthermore, learning disabilities and mood and anxiety disorders are common in patients with TS (Jurič et al., 2016). The comorbidities of TS and other psychiatric disorders suggest that the etiology and pathophysiology of TS may be universally applicable to a wide range of psychiatric diseases.

TS is most likely caused by a variety of genetic and environmental factors and presents with obvious genetic heterogeneity (Liu et al., 2020). Evidence based on familial aggregation studies showed that the risk for first‐degree relatives is significantly higher than that for individuals in the general population (Pauls et al., 1981; Pauls et al., 1991). In twin studies, 53−56% monozygotic twins were concordant for TS, whereas only 8% of dizygotic twins were concordant for TS (Liu et al., 2020; Price et al., 1985). Although these studies indicate that genetic factors play a significant role in TS etiology, the exact genetic risk remains unknown. TS is polygenic, involving multiple common risk variants accompanied by rare, inherited, or de novo mutations. Genome‐wide, candidate gene, and CNV studies on TS etiopathogenesis have revealed multiple gene variants, including dopaminergic (*DRD2,DRD4,DAT1*) (Díaz‐Anzaldúa et al., 2004; Herzberg et al., 2010; Tarnok et al., 2007), serotonergic (*HTR1A, HTR2C*) (Dehning et al., 2010; Lam et al., 1996), glutamatergic (*SLC1A3*) (Adamczyk et al., 2011), synapse developmental and functional (*SLITRK1*, *NLGN4*, and *NRXN1*) (Abelson et al., 2005; Lawson‐Yuen et al., 2008; Nag et al., 2013), and neurotransmitter receptor (*GRIN2b*, *HDC*) (Ercan‐Sencicek et al., 2010). However, because of the small sample size, the restricted number of variants in each study, and the inherent difficulties of risk‐gene studies involving genetically heterogeneous disorders, no individual candidate gene has met the statistical criteria of TS risk factors. Nevertheless, these potential genes might provide clues to the neurobiology of TS.

The histidine decarboxylase gene (*HDC)* has been the focus of TS research in recent years. We have investigated the role of the histidine decarboxylase gene (*HDC*) in TS susceptibility in the Chinese Han population (Dong et al., 2016), but the findings indicate an unlikely association between *HDC* and TS in the Chinese Han population. Here, we first introduce the discovery of *HDC* and the mutations that confer susceptibility to TS. Then the significance and difficulties of using *HDC*‐knockout (KO) mice as a pathophysiological model of TS are discussed. Subsequently, recent research using animal models is summarized from two different aspects of *HDC*‐KO mice: the change of dopamine and its receptor and the change of histamine and its receptor. Finally, we express our opinion about the possible brain nerve cell changes in *HDC*‐KO mice and their potential roles in TS. These findings highlight the importance of *HDC*‐KO mice for TS research and provide a new perspective for future research. We believe that our study makes a significant contribution to the literature because we demonstrate the role of the *HDC‐*KO mouse model in enhancing the understanding of TS pathophysiology, which is an important area for future research with direct implications for the clinical management of selected phenotypes.

### 1.1 Mutations in *HDC* confers susceptibility to TS

*HDC* is a member of the group II decarboxylase family that encodes L‐histidine decarboxylase and forms a homodimer that converts L‐histidine to histamine in a pyridoxal phosphate‐dependent manner (Dong et al., 2016). Ercan‐Sencicek et al. (2010) conducted a genome‐wide analysis of DNA samples from a two‐generation pedigree and identified the complete linkage of a 3.4‐centimorgan (cM) segment of chromosome 15 with an LOD score of 2.05. Polymerase chain reaction (PCR) assay was then conducted to amplify and sequence the exons and intron–exon junctions of 51 known genes within this linkage interval. A rare terminating mutation was identified in the exon 9 of *HDC*, namely the *W317X* mutation. The enzymatic activities of the *W317X* mutant, wild‐type *HDC*, and the combination of different proportions of the *W317X* mutant with a fixed number of wild‐type *HDC* were evaluated via transcriptional and translational assays in vitro. The dose–response characteristics of histamine indicated the dominant‐negative effects of mutant proteins, in line with the finding that the *W317X* mutant decreases histamine content in the central nervous system (Ohtsu et al., 2001).

Ercan‐Sencicek et al. first reported that mutations in *HDC* confer susceptibility to TS, underlying its significance in the molecular mechanism of TS. Subsequently, CNVs have revealed the enrichment of genes within histaminergic signaling pathways in a case‐control study involving 460 patients with TS and 1131 healthy individuals (Fernandez et al., 2012). Furthermore, Karagiannidis et al. (2013) showed overtransmission of alleles for rs854150 and rs1894236 in the *HDC* region in a large sample of 520 families from seven European countries, suggesting that rs1894236 may be directly involved in the transcriptional regulation of *HDC*. Taken together, these studies indicated that *HDC* confers susceptibility to TS, supporting the hypothesis that histamine dysregulation is strongly associated with TS.

### 1.2 *HDC‐*KO mice as a pathophysiological model of TS

Because of the solid evidence for the role of histamine dysregulation in neuropsychiatric disorders, *HDC*‐KO mice have received increasing attention. *HDC−*/− and heterozygote mice show increased tic‐like stereotypies and D_2_+D_3_ receptor dysregulation, recapitulating the core phenomenology of TS. Furthermore, preconditioning with haloperidol or injection with histamine alleviates the stereotypies of *HDC*‐KO mice, indicating that the behavioral and neurochemical abnormalities are similar to patients with TS with *HDC W317X* mutations (Baldan et al., 2014). These findings demonstrate the validity of *HDC*‐KO mouse model for TS. One of its weakness is that stereotypies occur after pharmacological challenges, complicating the use of *HDC−*/− mice to discover new therapies (Xu et al., 2015). Histamine‐deficient mice treated with a stimulant present persistent and progressive enhancement of locomotor and stereotypic behaviors, such as sniffing, biting, and rearing, and has thus been proposed as a model of human tics (Kubota et al., 2002). However, Xu et al. (2015) induced tic‐like stereotypes in the *HDC−*/− model stimulated by cued fear conditioning, suggesting that the stress‐induced stereotypy phenotype is more suitable for future pharmacological studies due to the presence of enhanced tic‐like behavior without pharmacological challenge(Baldan et al., 2014).

Studies on neuropsychiatric disorders are hampered by the neurobiology of the brain and the ethical or practical difficulties presented by invasive technologies. There are limitations in our recognition of details of the molecular biology and physiology in the human brain, although noninvasive technologies to study the structure and function of the human brain are being developed rapidly (Nestler & Hyman, 2010). The use of highly explicit and rare mutations to establish animal models has an important value for the study of the pathophysiology of TS. However, differences in species prevent the full replication of the core traits of patients with neuropsychiatric disorders, such as reading and thinking, as these are unique to humans. The key characteristics of the disease reproduced in animal models may not be specifically identified and quantified; therefore, trying to study all aspects of the disease in animal models is impractical (Pittenger, 2020). Although some important aspects of TS in *HDC*‐KO mice, such as repetitive, tic‐like behaviors, can be observed and quantified, it is impossible to assess whether they are associated with the premonitory urges that characterize tics (Pittenger, 2017). Our knowledge of the pathophysiology of TS is still limited. However, the corticostriatal circuits in mice and humans are broadly similar in tic disorders, and multiple levels of parallelism have been established between *HDC‐*KO mice and human *HDC W317X* mutation (Pittenger, 2020). Therefore, a careful study of pathophysiological processes in mice may shed light on the pathogenesis of human diseases.

### 1.3 Changes in dopamine, histamine, and their receptors in HDC‐KO mice

#### 1.3.1 Changes in dopamine and dopamine receptors

Previous studies have suggested that histamine (HA) regulates dopamine (DA) negatively (Haas et al., 2008; Schlicker et al., 1994). A recent study shows that the HA level of *HDC−*/− mice is significantly decreased, whereas that of DA is significantly increased (Baldan et al., 2014). The striatal DA turnover in *HDC‐*KO mice is increased (Dere et al., 2003). Baldan et al. (2014) used positron emission tomography to examine DA receptors in vivo because DA cannot be directly detected in humans, and the compensatory changes in the dopamine receptors reflect the maladjustment of dopaminergic regulation within the basal ganglia. They found that the dopamine D_2_+D_3_ receptor within the basal ganglia is dysregulated in patients with TS having the *W317X* mutation (Baldan et al., 2014). They focused on the dopamine D_2_+D_3_ receptors because D_2_ antagonists are the most effective pharmacotherapy for TS, and dopamine D_3_ receptors (D_3_R) may function as inhibitory autoreceptors (Jurič et al., 2016). Decreased striatal D_2_+D_3_ receptor binding and increased substantia nigra D_2_+D_3_ receptor binding may indicate a cellular response to a chronic increase in striatal DA (Stanwood et al., 2000). Elevated dopamine receptors in the substantia nigra were found in patients with mutated *HDC* and *HDC*‐deficient mice, further supporting the disorder of DA in vivo. These studies were the first to demonstrate the direct relationship between the change in histaminergic nerve transmission and the dopaminergic regulation of basal ganglia neural circuits in humans (Baldan et al., 2014).

Striatum medium spiny neurons (MSNs) can be divided into D1 receptor‐expressing (dMSNs) and D_2_ receptor‐expressing (iMSNs) ones. Striatonigral neurons expressing the D_1_ dopamine receptor (D_1_R) and striatopallidal neurons expressing the D_2_ dopamine receptor (D_2_R) provide excitatory and inhibitory feedback to the cortex, respectively. The dynamic imbalance between the two pathways is considered core to the TS pathogenesis (Albin & Mink, 2006; McBride & Parker, 2015). Elevated DA levels in *HDC*‐KO mice may affect the signal transduction of D_1_ R and D_2_R. As a rare TS pathophysiological model, *HDC−*/− mice need to characterize the imbalance in MSN signal (Baldan et al., 2014). Ak‐thymoma protein kinase (Akt) is widely expressed in iMSNs; this is confirmed using western blotting and immunohistochemistry as the reduction in pakt‐t308 positive cells in *HDC*‐deficient mice. Mitogen‐activated protein kinase (MAPK) pathway is activated in striatal dMSNs. MAPK is phosphorylated by rsk‐mediated s235/236 rpS6, and the number of phosphorylated rps6‐s235/236‐positive cells increase in the dorsal striatum of *HDC*‐KO mice (Baldan et al., 2014).

In addition, mTOR signaling remains unchanged and can be regulated by DA in both dMSNs and iMSNs. These cellular effects help to elucidate the striatal signaling abnormalities in *HDC*‐KO mice and identify new potential targets for the treatment of tic disorders (Baldan et al., 2014).

#### 1.3.2 Changes in histamine and histamine receptor

Histamine is an important monoamine neurotransmitter in the brain. The neuronal histaminergic system plays a central role in many basic physiological functions, such as the circadian rhythm, neuroendocrine homeostasis, energy metabolism, stress, cognition, sensory and motor function, attention, and memory. Neuronal histamine is synthesized in the tuberomammillary nucleus (TMN) in the posterior hypothalamus, and histamine‐positive fibers are projected into the cortex, hippocampus, basal ganglia, thalamus, and almost all other areas of the central nervous system (Haas et al., 2008). Histamine binds to four different G‐protein coupled receptors, known as the histamine H_1_ receptor (H_1_R), histamine H_2_ receptor (H_2_R), histamine H_3_ receptor (H_3_R), and histamine H_4_ receptor (H_4_R), located presynaptically (H_3_R and H_4_R) and postsynaptically (H_1_R, H_2_R, and H_3_R) to perform a range of regulatory functions. The changes in histamine levels in the brain are closely related to the central nervous system (Haas et al., 2008; Kárpáti et al., 2019).

Histamine was not detected in the striatum of *HDC−*/− mice, which indicates a chronic deficiency (Baldan et al., 2014). To make the effect of histamine deletion more pronounced, histaminergic neurons in the TMN of the hypothalamus of normally developing mice were specifically ablated or chemically silenced, lacking neurons or peripheral histamine throughout the process. Results showed that the ablation‐group mice have increased grooming and grooming times, slightly lower anxiety, and increased tendency of fear, but the exploratory movement and shock pulse before inhibition (PPI) were not changed. Further, the grooming of the histaminergic neuron inhibition group was also significantly increased. These results support the key role of TMN in regulating repetitive behavior (Rapanelli Frick, Bito et al., 2017). Moreover, after TMN inhibition, the striatum and cortex activities in the brain of mice were enhanced and the activation of dorsal striatum neurons after TMN inactivation led to significant grooming (Rapanelli, Frick, Bito et al., 2017). After the injection of histamine into the brain of the histaminergic neuronal inhibitory‐group mice, the movement was decreased, and the elevated grooming was reversed, which was similar to the conclusion of studies where the stereotypic behavior was produced by the activation of histaminergic signaling in the dorsal striatum of the *HDC*‐KO mice (Baldan et al., 2014; Rapanelli, Frick, Bito et al., 2017). These confirmed that acute histamine deficiency mediates these behavioral effects, and that pathogenesis is acute.

The expression of H_1_R mRNA in the striatum of *HDC*‐KO mice was quantitatively determined using in situ hybridization. The expression of H_1_R mRNA did not change significantly, and another mRNA expression study showed no significant increase in its expression in the hippocampus of *HDC−*/− mice (Frick et al., 2016; La Piana et al., 2012). In another study, radiation ligand binding and in situ hybridization were used to detect the H_2_R in *HDC*‐knockout mice, and they found that H_2_R mRNA was not altered in the striatum (Rapanelli, Frick, Pogorelov et al., 2017). In contrast, mRNA detection in the striatum homogenate of *HDC*‐deficient mice via real‐time reverse transcription‐PCR (RT‐PCR) showed a decrease in the H_4_R mRNA level (Frick et al., 2016). Iba1 staining was used to demonstrate that H_4_R antagonist blocks the effect of HA on microglia in *HDC−*/− mice. Ferreira et al. (2012) showed that the lipopolysaccharide (LPS)‐induced microglial migration and the production of interleukin‐1β (IL‐1β) are inhibited by histamine via H_4_R activation. Furthermore, HA can induce the activation of microglia and mitochondrial dysfunction in microglia via H_4_R‐MAPK (Dong et al., 2014). These results suggest that the H_4_ receptor is the main medium for histamine‐mediated microglia regulation.

Recent studies suggest that the dysregulation of the H_3_ receptor in the basal ganglia leads to the related phenomenology of tics in *HDC*‐KO mice (Pittenger, 2020). H_3_R has been presented as a potential and important regulator of signal transduction in MSNs (Moreno et al., 2011). Therefore, the H_3_ receptor has become the current focus of pathophysiology studies and a potential therapeutic target for tic disorders and related diseases (Pittenger, 2020). Using radiation ligand binding and in situ hybridization, we detected upregulated H_3_R in *HDC*‐KO mice. H_3_ receptors have high endogenous signal activity and can regulate intracellular signals in the absence of agonists (Morisset et al., 2000). Although *HDC−*/− mice lack histamine, H_3_R may regulate the striatum function. H_3_R agonists RAMH and immepip do not change the movement or stereotypes in wild‐type mice or significantly change the level of striatal DA in wild‐type mice (Alfaro‐Rodriguez et al., 2013). In contrast, in *HDC*‐KO mice, RAMH and immepip exhibit stereotypes in a dose‐dependent manner, leading to a modest but significant increase in striatum DA, as opposed to the negative regulation of DA release by H_3_R in in vitro studies (Schlicker et al., 1994). In addition, the H_3_R antagonist JNJ5207852 can block this stereotype after the use of RAMH and immepip, further confirming the effects of H_3_R (Rapanelli, Frick, Pogorelov et al., 2017). These reports were the first to demonstrate a direct relationship between H_3_R activation and tic‐like phenomenology in a pathophysiology‐based TS model (Rapanelli, Frick, Pogorelov et al., 2017).

The majority of H_3_R in the striatum is postsynaptic, which cascades with signals from intracellular striatal MSNs in a complex and cell‐type‐specific manner (Ferrada et al., 2008; Ferrada et al., 2009; Moreno et al., 2011; Rapanelli et al., 2016). In vitro studies have demonstrated that H_3_R can regulate MAPK signaling via heterodimerization with dopamine D_2_ receptor (Ferrada et al., 2008; Ferrada et al., 2009; Moreno et al., 2011). Previous studies have shown that both H_3_R mRNA expression and radio‐ligand binding are upregulated in the striatum of *HDC−*/− mice (Rapanelli, Frick, Pogorelov et al., 2017).

**FIGURE 1:**
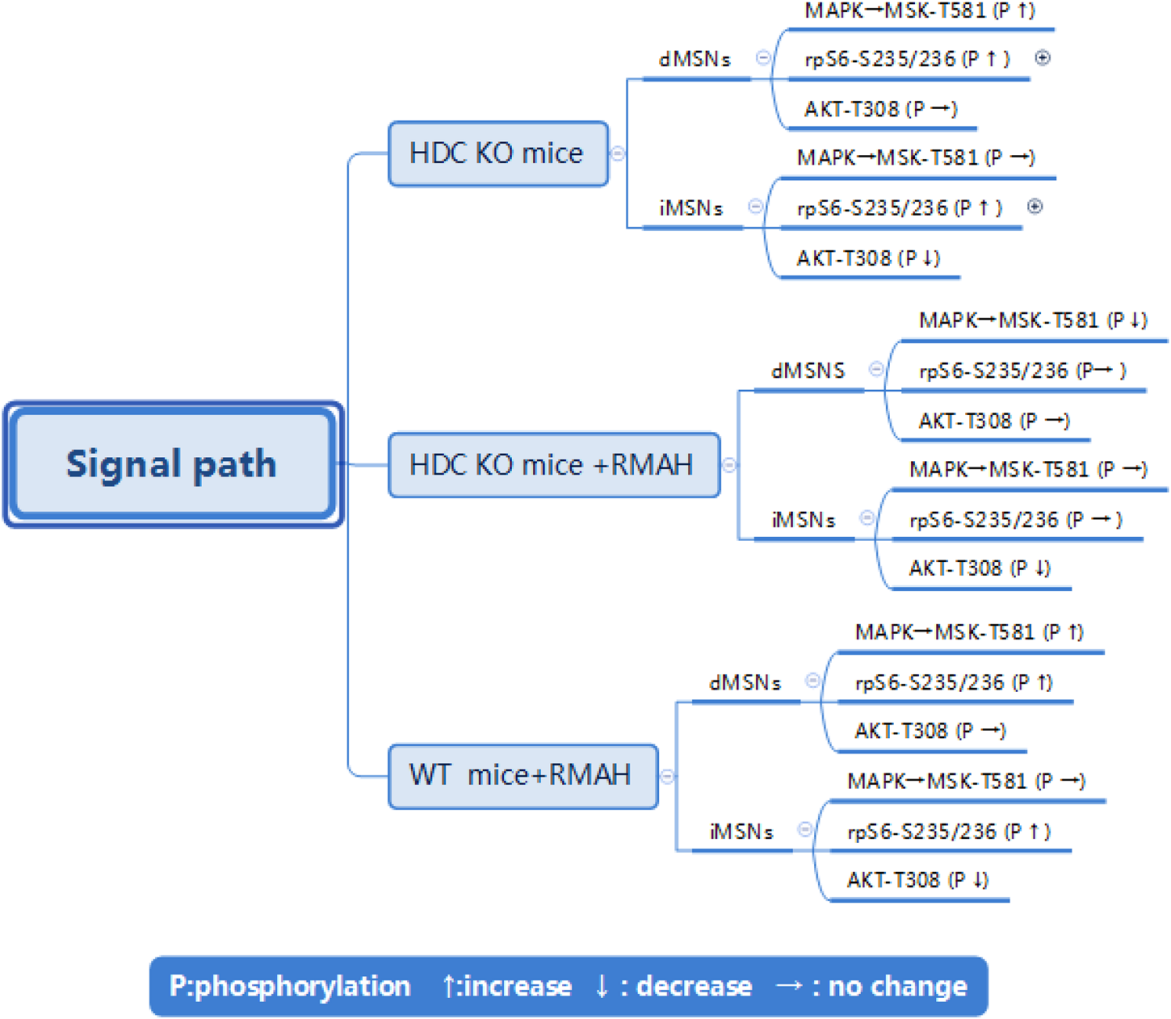
The changes in intracellular signals of dMSNs and iMSNs in the dorsal striatum of *HDC*‐KO mice compared with the intracellular signals of dMSNs and iMSNs at baseline in WT mice. *HDC*‐KO and WT mice were treated with RAMH

Compared with the intracellular signals of dMSNs and iMSNs at baseline in wild‐type (WT) mice, a recent study analyzed the different effects of H_3_R and its agonists on the intracellular signals of dMSNs and iMSNs in the dorsal striatum of mice (Rapanelli et al., 2018; Rapanelli et al., 2016) (Figure 1). Particularly, they found that in *HDC*‐KO mice, MAPK signaling is activated at baseline, and MSK phosphorylation is activated at T581 of MAPK in dMSNs. In iMSNs, MSK‐T581 phosphorylation does not change at the baseline. RpS6 phosphorylation at s235/236 increases in dMSNs and iMSNs at the baseline. The phosphorylation at T308 of Akt decreases in iMSNs, whereas that of Akt‐T038 in dMSNs stays unchanged at the baseline (Rapanelli et al., 2018).

In *HDC*‐KO mice treated with RAMH, MAPK signal is activated in dMSNs. Phosphorylation of MSK‐T581 decreases in dMSNs, whereas that of MSK‐T581, the target of MAPK, is not affected in iMSNs. RAMH has no significant effect on rpS6 phosphorylation at s235/236 but decreases the phosphorylation of AKT‐T308 in iMSNs. Further, RAMH has no significant effect on the phosphorylation of AKT‐T308 in dMSNs (Rapanelli et al., 2018) (Figure 1).

In WT mice treated with RAMH, MSK phosphorylation at MAPK target T581 in dMSNs is higher than that of *HDC‐*KO mice treated with RAMH, but the phosphorylation of MSK‐T581 is not affected in iMSNs. There is a significant increase in the phosphorylation of rps6‐S235/S236 in dMSNs and of rpS6‐S235/236 in iMSNs. Phosporylation of AKT‐T308 in iMSNs is reduced, whereas that of AKT‐T308 in dMSNs is unaffected (Rapanelli et al., 2018) (Figure 1).

The baseline signal abnormalities found in *HDC*‐KO mice are similar to the results of H_3_R activation in WT mice. Particularly, in the MAPK signaling pathway in dMSNs, the baseline phosphorylation of MSK at T581 in *HDC*‐KO mice is similar to that in WT mice stimulated by RAMH (Baldan et al., 2014). The phosphorylation of rpS6 at S235/236 in dMSNs and iMSNs and dephosphorylation of AKT‐T308 in iMSNs are similar in *HDC−*/− and WT mice. Therefore, the constitutive signaling of upregulated H_3_ receptors can mediate the observed alterations at baseline in MSN signaling and can explain many of the molecular and behavioral abnormalities in *HDC‐*KO mice despite the absence of HA. H_3_R affects the balance of D_1_‐MSN and D_2_‐MSN active pathways, and the treatment of H_3_R agonist exacerbates the imbalance. The imbalance in these pathways in the striatum may cause the tics (Albin & Mink, 2006; McBride & Parker, 2015; Rapanelli et al., 2018). However, the MAPK signaling and MSK‐T581 phosphorylation are activated at the baseline in dMSNs of *HDC‐*KO mice. The phosphorylation of MSK‐T581 is reduced in dMSNs of *HDC−*/− mice treated with RAMH. The upregulated receptors reverse the intracellular signaling in *HDC*‐KO mice treated with RAMH, which is an important direction for future studies on *HDC−*/− mice (Rapanelli et al., 2018). These molecular abnormalities are best understood in the context of previous behavioral work, for which Rapanelli, Frick, Pogorelov et al. (2017) found RAMH treatment to produce stereotypies in the *HDC*‐KO mouse. Previous studies revealed that the elevated stereotypic behavior is also seen in these animals after amphetamine challenge, indicating that they have a generalized instability of the striatal network that predisposes to repetitive behavioral pathology (Baldan et al., 2014; Pittenger, 2017). The abnormalities at baseline in striatal signaling documented here may represent the mechanistic correlation of this predisposition (Figure 1).

### 1.4 Changes in nerve cells in the brain of *HDC*‐KO mice

#### 1.4.1 Neuron

Striatal interneurons (SI) are key players in controlling excitatory inputs in the cortex and thalamus, thus maintaining the excitatory‐inhibitory balance in the striatum (Lim et al., 2014; Tepper et al., 2010). The types of SI include the GABAergic‐ and cholinergic choline acetyltransferase (ChAT)‐positive subgroups. The abnormalities of parvalbumin (PV) and nitric oxide synthase (NOS) containing GABAergic and ChAT SI are associated with TS. Immunohistochemical studies performed on TS subjects have revealed decreased numbers of PV SI in the caudate nucleus and altered distribution of PV neurons in the globus pallidus (GP) (Kalanithi et al., 2005; Kataoka et al., 2010). Furthermore, a postmortem study on TS patients showed lower numbers of immunopositive ChAT SI in associative and sensorimotor parts of the caudate nucleus and putamen (Kataoka et al., 2010; Lennington et al., 2016). Lennington et al. (2016) also found decreased number of NOS interneurons in the striatum of TS patients. Recently, Abdurakhmanova et al. investigated whether *HDC*‐KO mice have the corresponding morphological and cytological changes in patients with TS. The brain anatomy and cell structure of *HDC−*/− mice have been studied via MRI, diffusion tensor imaging, stereology, and immunohistochemistry.

**FIGURE 2:**
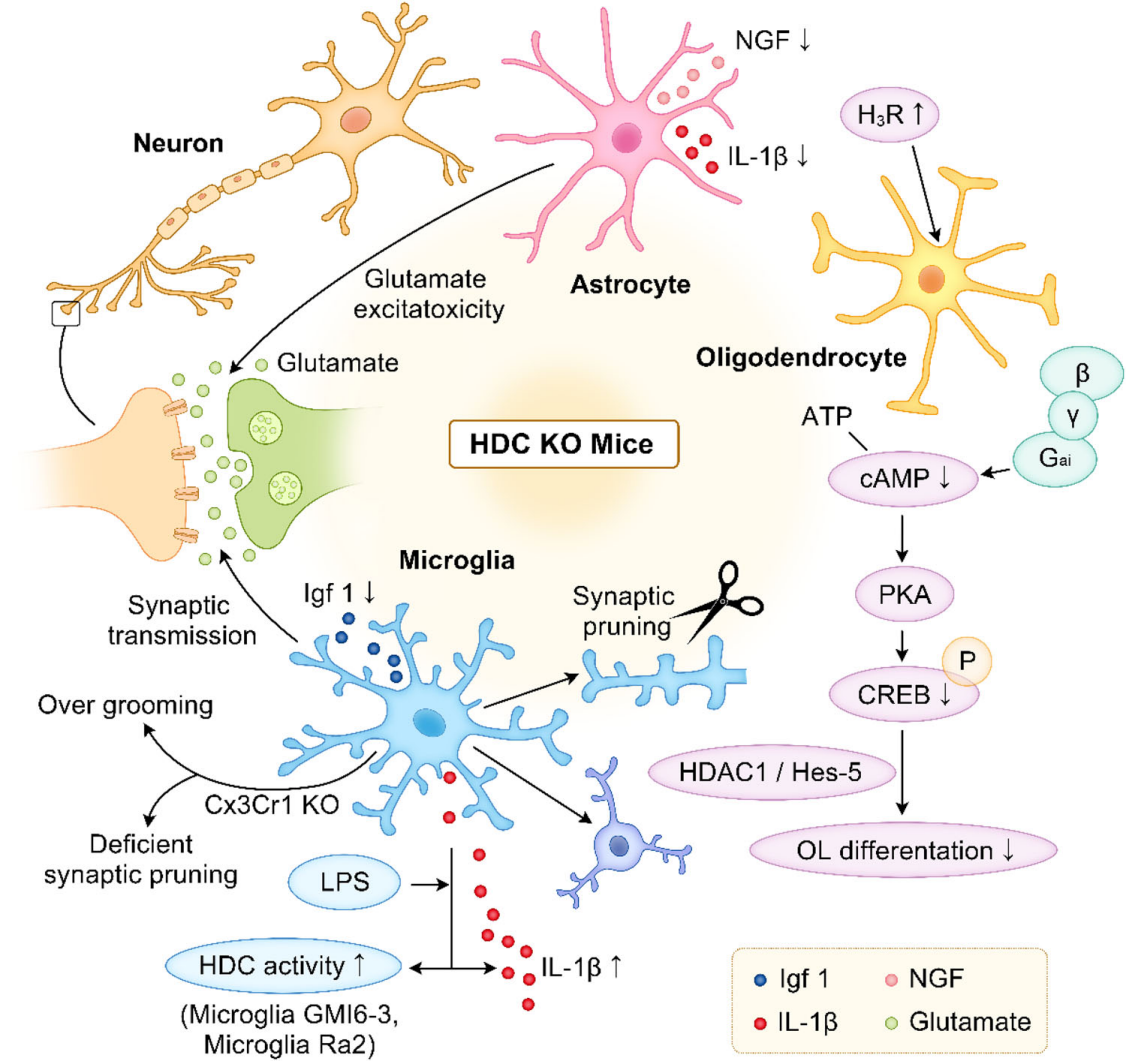
Possible mechanisms of abnormal microglia, oligodendrocyte, and astrocyte functions in HDC‐KO mice

When *HDC−*/− and WT mice were compared, there was no difference in the volume of the whole brain, cortex, and striatum, nor in the number of intrastriatal neurons. In addition, stereological estimation of cortical GABAergic interneurons number showed no significant difference. In *HDC*‐KO mice, the cortical cell structures were well‐preserved and the number of GABAergic neurons in GP did not change. This study indicated that *HDC* deficiency in mice does not interfere with the development of striatal neurons and intercortical neurons, nor do they result in the morphological and cytological phenotypes that are characteristic for patients with TS. Thus, the behavioral changes caused by histamine deficiency may be due to an imbalance of neurotransmitters at the striatum level (Abdurakhmanova et al., 2017) (Figure 2).

#### 1.4.2 Microglia

Microglia are the innate immune cells of the central nervous system that play an important role in immune surveillance. They are important cells in adult neurogenesis that support the survival of neurons. Because of recent studies, microglia have taken on new roles in brain development, homeostasis, and plasticity (Paolicelli et al., 2011; Ziv et al., 2006). Particularly, microglia prune synapses, which is necessary to form brain circuits and normal connections during normal development in mice (Ji et al., 2013; Zhan et al., 2014). Microglial dysregulation has also been observed in TS. Immunohistochemical studies on the postmortem brain of TS patients showed an elevated count of cells of the macrophage/microglia lineage and activated morphological features in the caudate. These results suggest that brain microglia activation might be the underlying mechanism in the inflammatory changes observed in the brain (Lennington et al., 2016). The role of histamine in the regulation of microglia is controversial. Ferreira et al. (2012) showed that HA may counter the proinflammatory activation of microglia as HA blocks LPS‐induced N9 cell (a microglia‐like cell line) motility and proinflammatory cytokine secretion. The microglia can express all histamine receptors (H1R, H2R, H3R, and H4R) constitutively. Dong et al. (2014) found that HA selectively activates the expression of H1R and H4R in the primary microglia culture in a dose‐dependent manner. Following microglia activation, the release of inflammatory cytokines (tumor necrosis factor [TNF]‐alpha and interleukin‐6 [IL‐6]) was upregulated in a dose‐dependent manner by histamine. The production of histamine‐induced TNF‐alpha and IL‐6 can be reduced by the H1R and H4R antagonists. Zhu et al. (2014) reported similar results in primary microglial cultures. These studies suggest that HA may have a proinflammatory effect.

An in vivo study investigated the effects of HA deficiency in HDC KO mice and of HA receptor stimulation in wild‐type animals via immunohistochemistry. Frick et al. (2016) observed the abnormal activation of microglia in *HDC*‐deficient mice with decreased microglia branches but found no change in the total number of microglia using anti‐iba1 immunohistochemistry. The selective depletion of histamine neurons in TMN by targeted ablation also occurred in the *HDC*‐KO model. Three days after the acute injection of histamine into the brain of WT mice, the number and branch density of microglia in the striatum of WT mice increased significantly. These results suggest that neurogenic HA may be responsible for the acute modulation of microglia cells. RT‐PCR was used to detect the expression of cytokines associated with inflammation and neuroprotection and revealed a decrease in insulin‐like growth factor 1 (igf‐1) of *HDC*‐KO mice. Igf‐1 has a neuroprotective and limiting effect on inflammatory responses and is an important mediator of homeostatic microglial function. Therefore, the decrease in igf‐1 expression in the microglia of *HDC*‐KO mice might lead to impaired neuroprotection and dysregulation of the response to proinflammatory stimuli, which contribute to enhanced susceptibility to neuroinflammation after an environmental challenge (Frick & Pittenger, 2016). LPS challenge triggers an exaggerated response in *HDC‐*KO mice, accompanied by enhanced microglial activation in the striatum and elevated secretion of proinflammatory cytokine IL‐1β. Frick et al. (2016) found that microglia in *HDC−*/− mice show increased sensitivity to LPS, and the effect of lipopolysaccharide on the increase in microglial cell density in *HDC*‐KO mice is dose‐dependent. Microglial abnormalities were also found in the hypothalamus of *HDC‐*KO mice, but not in the motor cortex, and such regional differences were not observed in the microglia culture experiments in vitro.

Microglia are phagocytes that infiltrate the brain during development and remodel synapses as the brain matures. Synaptic pruning by microglia is necessary for the formation of normal connectivity and brain circuitry (Schafer et al., 2012). A transient reduction in microglia during the early postnatal period and a deficient synaptic pruning in Cx3cr1 (expressed by microglia in the brain) have been observed in KO mice, which resulted in changes in the neuron‐microglia communication. The deficient synaptic pruning is associated with repetitive behavioral phenotypes, deficits in social interaction, weak synaptic transmission, and decreased functional brain connectivity, which are thought to be associated with neurodevelopmental and neuropsychiatric disorders (Zhan et al., 2014). Frick and Pittenger (2016) found that Cx3cr1‐KO mice present with excessive grooming, similar to *HDC*‐KO mice. As the deficiency in microglia‐mediated synaptic pruning may lead to neurodevelopmental and neuropsychiatric disorders, it is necessary to study synaptic pruning in *HDC*‐KO mice and other TS mouse models.

Niimi et al. (1997) reported that HDC activity in the primary culture of rat diencephalon cells with no mast cells can be upregulated by LPS and IL‐1β. In addition, our preliminary in situ hybridization studies with primary brain cultures of rat embryos showed that microglia‐like cells were positive for HDC mRNA. To investigate whether microglia are the third compartment of histamine production in the brain, the *HDC* activity of microglia GMI 6‐3 and microglia Ra2 in the presence or absence of LPS has been studied (Katoh et al., 2001). *HDC* activity increases after LPS stimulation in the microglia GMI 6‐3 of mice. In addition, Northern blot analysis showed that the *HDC* mRNA expression is induced in microglia GMI 6‐3 treated with LPS, showing that certain microglial types in the brain can produce histamine. These studies indicate that histamine in the brain has a close and complex relationship with microglia. Therefore, the dysregulation mechanism of microglia plays an important role in the pathophysiology of *HDC*‐KO mice (Figure 2).

#### 1.4.3 Oligodendrocyte

In the past two years, the H_3_ receptor (H_3_R) has received increasing research attention. Some studies have showed that the imbalance in H_3_R in the basal ganglia leads to the correlational phenomenology in *HDC−*/− mice. The H_3_R in the striatum of HDC‐KO mice was upregulated and still had constitutive activity without the ligand (Morisset et al., 2000; Pittenger, 2020). H3R, a member of the histamine receptor family, is highly expressed in the central nervous system and has been identified as a potential drug target for the treatment of neurological and psychiatric diseases, but its function in oligodendrocytes remains unknown.

In a 1000‐drug screening trial, Chen et al. have identified seven H_3_R antagonists that promoted the differentiation of oligodendrocyte precursor cell (OPC) in a concentration‐dependent manner. To identify their targets, OPC was treated with H_3_R reverse agonist and medium antagonist. The results showed that the total number of OPC cells is not changed, indicating that both the H_3_R reverse agonist and medium antagonist had no effect on OPC proliferation. Although medium antagonists had no effect on OPC differentiation, H_3_R reverse agonists promoted OPC differentiation in a dose‐dependent manner. This difference suggests that H_3_R reverse agonist positively regulates OPC differentiation. Western blot analysis showed that H_3_R is moderately expressed in oligodendrocytes, which decreased on the first day of OPC differentiation, but was upregulated on the third and fifth days and throughout the differentiation process. H_3_R knockout resulted in the increased expression of myelin related glycoprotein (MAG) and myelin basic protein, markers of oligodendrocyte differentiation, whereas its overexpression resulted in their low expression and decreased number of mature oligodendrocytes. Therefore, increasing H_3_R expression in the absence of histamine is enough to inhibit the differentiation of oligodendrocytes. These findings suggest that the constitutive activity of H_3_R negatively regulates the differentiation of oligodendrocytes (Chen et al., 2017).

Studies have shown that the differentiation of oligodendrocytes is regulated by cyclic adenosine monophosphate (cAMP)and cAMP‐response element binding protein (CREB), and that the H_3_R reverse activator GSK247246 increases the intracellular level of cAMP and the phosphorylation of CREB, which provides evidence for the correlation between H_3_R and cAMP/CREB activity during the differentiation of oligodendrocytes (Chen et al., 2017; Raible & McMorris, 1993; Sato‐Bigbee & DeVries, 1996). These results are consistent with another study confirming that the H_3_R activity of histaminergic neurons in rodent brains is regulated by the cAMP signal. We speculate that demyelinating disease or demyelination symptoms may occur in HDC‐KO knockout mice. The changes in and differentiation mechanisms of oligodendrocytes in HDC‐KO knockout mice and the association with other demyelinating diseases may be worth exploring (Morisset et al., 2000) (Figure 2).

#### 1.4.4 Astrocytes

Astrocytes play an important role in the physiological functions of the central nervous system. They provide nutritional support for migrating neurons and guidance for the formation and maintenance of neural pathways. Furthermore, astrocytes participate in the control of cerebral blood flow, energy metabolism, and ionic constant pressure, as well as in synaptic formation and maturation. Previous research suggests that astrocytic signals play an important role in complex rodent behaviors, such as memory and circadian time‐keeping (Brancaccio et al., 2017; Santello et al., 2019; Yu et al., 2018). Recent genome‐wide association analysis showed that TS is associated with a set of astrocyte genomes, mainly involving astrocyte carbohydrate metabolism. TS is primarily associated with the astrocyte‐neuron metabolic coupling (ANMC) subgenome, which contains 33 genes that encode enzymes or transporters involved in glycolysis or glutamine metabolism. Genetic variations in glycolysis and glutamate metabolism have profound effects on astrocytes regulating synaptic function. These findings indicate that the process of astrocyte‐neuron metabolic coupling may be an important contributor to TS pathogenesis (de Leeuw et al., 2015). Glutamate is the most important excitatory neurotransmitter in the central nervous system, but excessive amounts of glutamate can activate glutamate receptors exceedingly, leading to the death of neurons, which is known as excitatory toxicity. Increasing evidence shows that glial cells in the brain, especially astrocytes, control the extracellular glutamate content and prevent excitatory toxicity. Due to the lack of extracellular metabolic pathways, glutamate, after being released into the synaptic cleft, is mainly taken up by glutamate transporters (GLT‐1, GLAST) in astrocytes and converted into intracellular glutamine by glutamine synthetase (GS). Subsequently, the glutamine is transported back to glutamate or γ‐aminobutyric acid (GABA) neurons as raw material for glutamate and GABA synthesis. Rodriguez et al. (1989) reported that the GS activity in cerebellar astrocytes can be elevated by histamine. Moreover, histamine can upregulate GLT‐1, which provides another key link in glutamate metabolism by astrocytes via H1 receptor, thus reducing extracellular glutamate levels and playing a neuroprotective role in excitotoxicity and ischemic injury (Hu & Chen, 2012; Hu et al., 2012).

A study has speculated that the glutamate‐glutamate cycle regulatory factor and glutamate transporters of astrocytes may function in histamine system protection and have a treatment effect via the removal of excess glutamate. This can reduce the excitatory toxicity in the early stages of cerebral ischemia, balance the glutamate synaptic transmission, and protect early neurons. However, further in vivo studies are needed to confirm this hypothesis. There is very little evidence for the interaction between histamine and astrocyte immunoregulation. A study has shown that histamine significantly amplifies the secretion of NGF (a neurotrophic factor) in astrocytes stimulated by the proinflammatory cytokine IL‐6 and has a superposition effect with the proinflammatory cytokine IL‐1β (Lipnik‐Stangelj, 2006). Histamine is an important player in the interaction between astrocytes and proinflammatory cytokines, which may increase not only neuronal inflammation but also NGF production to counteract neuronal inflammation. Histamine plays an important role in astrocyte activity, such as energy metabolism and immune response. Therefore, we believe that it plays an important role in regulating astrocyte function, which deserves further investigation using *HDC‐*deficient mice (Jurič et al., 2016) (Figure 2).

## 2 CONCLUSIONS

In summary, TS is a developmental neuropsychiatric disorder associated with genetic factors in most cases. *HDC* variation plays an important role in genetic susceptibility to TS. Subsequently, *HDC‐*KO mice is a good animal model that contributes to substantial progress in the understanding of TS pathophysiology. The parallel TS‐related behavioral and neurochemical abnormalities in humans with *HDC W317X* mutation and *HDC*‐KO and heterozygous mice provide favorable evidence for the causal association between *HDC* deficiency and TS. The observed basal ganglia abnormalities in *HDC*‐deficient mice, which involve the histaminergic regulation of dopaminergic modulation and information processing, are important factors in TS pathogenesis. Furthermore, we also focused on the possible alterations in the information processing of nerve cells in the brain of *HDC*‐KO mice, which contribute to pathophysiological changes associated with TS. Future studies on histaminergic regulation and dopamine modulation of striatal circuits and their potential role in nerve cells will be important to further our understanding of brain mechanisms relevant to TS.

## CONFLICT OF INTEREST

All authors claim that there are no conflicts of interest.

## AUTHOR CONTRIBUTIONS

SL and XZ designed the project conception. LX, CZ, MZ, FC, and CG performed the literature search and analysis. LX wrote the manuscript with contribution from SL and XZ. All authors read and approved the final manuscript.

### PEER REVIEW

The peer review history for this article is available at https://publons.com/publon/10.1002/brb3.2511

## Data Availability

Data sharing is not applicable to this article as no new data were created or analyzed in this study.
